# Circulating Cell-Free DNA as Biomarker of Taxane Resistance in Metastatic Castration-Resistant Prostate Cancer

**DOI:** 10.3390/cancers13164055

**Published:** 2021-08-12

**Authors:** Edoardo Francini, Fang-Shu Ou, Justin Rhoades, Eric G. Wolfe, Edward P. O’Connor, Gavin Ha, Gregory Gydush, Kaitlin M. Kelleher, Rupal S. Bhatt, Steven P. Balk, Christopher J. Sweeney, Viktor A. Adalsteinsson, Mary-Ellen Taplin, Atish D. Choudhury

**Affiliations:** 1 Department of Experimental and Clinical Medicine, University of Florence, 50134 Florence, Italy; 2 Dana-Farber Cancer Institute, Harvard Medical School, Boston, MA 02215, USA; edwardpoconnor@gmail.com (E.P.O.); gavinha@broadinstitute.org (G.H.); kmakelleher@gmail.com (K.M.K.); christopher_sweeney@dfci.harvard.edu (C.J.S.); Mary_Taplin@DFCI.harvard.edu (M.-E.T.); aChoudhury@partners.org (A.D.C.); 3 Department of Quantitative Health Sciences, Mayo Clinic, Rochester, MN 55905, USA; Ou.Fang-Shu@mayo.edu (F.-S.O.); EricWolfe1@aol.com (E.G.W.); 4 Eli and Edythe L. Broad Institute of MIT and Harvard, Cambridge, MA 02142, USA; rhoades@broadinstitute.org (J.R.); greggydush@gmail.com (G.G.); viktor@broadinstitute.org (V.A.A.); 5 Beth Israel Deaconess Medical Center, Boston, MA 02215, USA; rbhatt@bidmc.harvard.edu (R.S.B.); sbalk@bidmc.harvard.edu (S.P.B.)

**Keywords:** cell-free DNA, biomarkers, metastatic castration-resistant prostate cancer, taxanes

## Abstract

**Simple Summary:**

There is a clinical need for biomarkers predictive of resistance to taxane therapy for patients with metastatic castration-resistant prostate cancer (mCRPC). Based on existing evidence for the role of ATP-binding cassette (ABC) transporters in taxane resistance, we sought to assess the association between *ABCB1* gene amplification and primary resistance to docetaxel or cabazitaxel in mCRPC patients using sparse whole genome sequencing from plasma-derived cell-free DNA (cfDNA). Because sparse whole genome sequencing is more cost-effective than traditional cfDNA profiling and less invasive than tissue biopsies, a clinically useful biomarker of taxane resistance discovered using this technique has the potential to be broadly applied in clinical practice. We did not detect a statistically significant association between *ABCB1* amplification detected by this method and docetaxel or cabazitaxel resistance in our cohort. Future studies with larger samples including *ABCB1* amplification in a suite of putative biomarkers are warranted to draw definitive conclusions.

**Abstract:**

There are no biomarkers predictive of resistance to docetaxel or cabazitaxel validated for patients with metastatic castration-resistant prostate cancer (mCRPC). We assessed the association between *ABCB1* amplification and primary resistance to docetaxel or cabazitaxel for patients with mCRPC, using circulating cell-free DNA (cfDNA). Patients with ≥1 plasma sample drawn within 12 months before starting docetaxel (cohort A) or cabazitaxel (cohort B) for mCRPC were identified from the Dana–Farber Cancer Institute IRB approved database. Sparse whole genome sequencing was performed on the selected cfDNA samples and tumor fractions were estimated using the computational tool ichorCNA. We evaluated the association between *ABCB1* amplification or other copy number alterations and primary resistance to docetaxel or cabazitaxel. Of the selected 176 patients, 45 samples in cohort A and 21 samples in cohort B had sufficient tumor content. No significant association was found between *ABCB1* amplification and primary resistance to docetaxel (*p* = 0.58; odds ratio (OR) = 1.49) or cabazitaxel (*p* = 0.97; OR = 1.06). No significant association was found between exploratory biomarkers and primary resistance to docetaxel or cabazitaxel. In this study, *ABCB1* amplification did not predict primary resistance to docetaxel or cabazitaxel for mCRPC. Future studies including *ABCB1* amplification in a suite of putative biomarkers and a larger cohort may aid in drawing definitive conclusions.

## 1. Introduction

Prostate cancer is currently the most diagnosed cancer, with 191,930 estimated new cases in 2020, and the second most common cause of cancer-related mortality in American men [1]. Most deaths occur when the disease progresses to metastatic castration-resistant prostate cancer (mCRPC) [2]. Currently, docetaxel and cabazitaxel are the standard chemotherapy options for mCRPC based on the results of four large phase 3 clinical trials [3,4,5,6]. Only approximately half of the patients receiving docetaxel for mCRPC have a biochemical response, while the rest have primarily resistance to docetaxel [3]. The rate of innate resistance to cabazitaxel for men with mCRPC progressing on docetaxel is higher at ~60% [5]. In addition, initially responding patients will progress in time. Several potential mechanisms of resistance have been investigated in the past, including the upregulation of multi-drug resistance (MDR) transporters [7] and the upregulation of phosphorylated AKT [8], with a potential predictive role for the detection of intranuclear androgen receptor splice variant 7 (AR-v7) in circulating tumor cells [9,10]; however, no biomarker of taxane resistance or response for patients with mCRPC has been validated for clinical decision-making. Finding biomarkers predictive of resistance or response to docetaxel and/or cabazitaxel would allow the clinician to use the most efficient drug, avoiding the administration of ineffective chemotherapy and thus sparing the patient unnecessary toxicity. Therefore, the discovery of response/resistance biomarkers is an important unmet clinical need.

The molecular mechanisms underpinning primary or acquired resistance to either taxane are not fully understood. Nonetheless, several studies reported a temporal correlation between the acquisition of docetaxel resistance and the upregulation of the Multidrug Resistance Protein 1 (MDR1) or ATP-Binding Cassette Sub-Family B Member 1 transporter (encoded by the *ABCB1* gene locus) in ovarian, breast, and prostate cancer [11,12,13]. The *ABCB1* gene encodes one of the major ATP-binding cassette (ABC) drug transporters, known for increasing drug efflux from tumor cells [12,13]. Among the other forms of genomic instability postulated as mechanisms underlying the increased expression of ABCB1, the amplification of this gene and its locus *7q21* was demonstrated in ovarian and breast cancer cells resistant to docetaxel [12]. As such, *ABCB1* amplification could also be potentially associated with taxane resistance in mCRPC. Other genetic alterations found associated with taxane resistance in pre-clinical studies of mCRPC were *ERG-TMPRSS2* translocation and deletion of *KDM5D*, a gene encoded on the Y chromosome [14,15,16]. These gene aberrations were frequently observed in patients with castration-resistant prostate cancer [17,18], and thus are also worth being evaluated as putative biomarkers of resistance to taxane therapy in mCRPC.

The Prostate Cancer Working Group (PCWG3) highlighted the importance of serial biologic tumor profiling using blood-based diagnostics to gain greater insight into the disease mechanisms of resistance and potentially detect clinically useful predictive biomarkers [19]. In this regard, plasma-derived circulating cell-free DNA (cfDNA) provides an accurate liquid tumor biopsy allowing for comprehensive tumor characterization including gene copy number alterations (CNAs) [20]. Previous analyses suggested the potential prognostic and predictive value of both the total quantity of cfDNA in the blood and the estimated tumor-derived portion of cfDNA (tumor fraction) [21,22]. 

We previously reported the utility of a computational tool ichorCNA, developed by investigators at the Broad Institute of MIT and Harvard, in estimating tumor fraction using 0.1× coverage whole genome sequencing (termed Ultra-Low Pass Whole Genome Sequencing, ULP-WGS) [22,23,24]. In addition to estimating tumor fraction, ichorCNA can also identify CNAs in cfDNA in a much more cost-effective manner than existing targeted sequencing platforms. In this study, we sought to assess the association of *ABCB1* amplification and primary resistance to docetaxel or cabazitaxel therapy for mCRPC. Given that ichorCNA does not identify translocations nor CNAs in chromosome Y, *ERG-TMPRSS2* fusions and *KDM5D* deletions cannot be detected through this method [23]. However, since ichorCNA allows for the assessment of the genome-wide copy number profile, a secondary endpoint was to explore the association of any other identified CNA and primary resistance to either taxane for mCRPC. Because it is often difficult to obtain metastatic tissue from patients for genomic testing, if successful, our method would provide a non-invasive biomarker predictive for docetaxel and/or cabazitaxel resistance, thus enabling an accessible and personalized approach to therapy management.

## 2. Materials and Methods

### 2.1. Study Population 

A cohort (A) of patients with at least 1 plasma sample collected and stored within 12 months prior to initiating docetaxel for mCRPC (between 2001 and 2016) and a cohort (B) of patients with at least 1 plasma sample collected and stored within 12 months prior to starting cabazitaxel for mCRPC (between 2010 and 2016) were identified from the Institutional Review Board approved Prostate Clinical Research Information System (CRIS) database at Dana–Farber Cancer Institute [25]. Patients who received cabazitaxel before docetaxel or any taxane in combination with other agents were excluded. All patients had consented to Dana–Farber/Harvard Cancer Center protocol no. 01-045 “Collection of Specimens and Clinical Data for Patients with Prostate cancer or at High Risk for Prostate Cancer”. This protocol allows for banking of tissue and blood specimens for research use, including comprehensive genetic sequencing and data sharing. 

### 2.2. Study Design

We analyzed the cfDNA of mCRPC patients who were treated with docetaxel and/or cabazitaxel and had plasma samples drawn and stored per protocol no. 01-045 within 12 months before treatment start. When multiple banked samples were available for a patient, we prioritized the sample that was collected closest to therapy initiation. The DNA isolated from banked plasma samples was subjected to ULP-WGS through ichorCNA to identify cases with sufficient tumor content to build sequencing libraries and detect *ABCB1* amplifications and any other CNA. The primary objective was to assess the correlation between *ABCB1* amplification (established biomarker) and primary resistance to docetaxel or cabazitaxel. The secondary objective was to evaluate the correlation between any detected CNA (exploratory biomarkers) and primary resistance to docetaxel or cabazitaxel. Primary resistance was defined as the absence of a response or death within 4 months from treatment initiation. In turn, response was defined as any of the following: (1) PSA decline ≥50% from baseline; (2) radiologic response according to RECIST criteria version 1.1 [26]. 

### 2.3. Study Procedures 

The identified banked plasma samples (1000 μL/subject) were retrieved from the genitourinary Gelb tumor bank. These frozen aliquots of plasma were thawed at room temperature and then subjected to high-speed spin. The Qiagen Circulating DNA kit (QIAGEN, Germantown, MD, USA) on the QIAsymphony liquid handling system was used to extract the cfDNA from the plasma samples. ULP-WGS was performed on the extracted cfDNA and sequencing information was run through ichorCNA to detect cases harboring detectable tumor DNA content and CNAs. In detail, the isolated cfDNA was quantified using the PicoGreen (Life Science Technologies, Waltham, MA, USA) assay on a Hamilton STAR-line liquid handling system. CfDNA sequencing libraries were constructed using the Kapa Hyper Prep kit with custom adapters (Integrated DNA Technologies, Coralville, IA, USA). A median of 5 ng of cfDNA input (3–20 ng) was used for ULP-WGS, which was performed using a Hamilton STAR-line liquid handling system (Hamilton Company, Reno, NV, USA). Constructed sequencing libraries were pooled (2 μL of each × 96 per pool) and sequenced using 100 bp paired-end runs over 1× lane on a HiSeq2500 (Illumina, San Diego, CA, USA) for ULP-WGS (~0.1× coverage). The genome was fractioned into T non-overlapping bins of 1 Mb and the HMMcopy Suite1 (http://compbio.bccrc.ca/softwar/hmmcopy/ (accessed on 12 August 2019)) tools were used to count aligned reads based on overlap within each bin. The read counts were then normalized to correct for GC-content and mappability biases using HMMcopy R package. Tumor copy number prediction and tumor DNA estimate were achieved using a hidden Markov Model. The software ichorCNA (available at https://github.com/broadinstitute/ichorCNA (accessed on 19 August 2019)) was used to derive genome-wide copy number plots. Samples passed a quality threshold (median absolute deviation score < 0.115) for accurate purity estimate. Considering the Broad Institute preliminary results [23], up to 40% of samples were estimated to yield >7% tumor purity, which was set as threshold to guarantee the quality of the data. As ichorCNA does not account for subclonal events, to guarantee accuracy, a gene was defined amplified or deleted when ≥5 copies or ≤1 copy, respectively, were found. The analyses were performed by Genomics Platform at the Broad Institute. In order to validate the output achieved with ichorCNA, GISTIC2.0 was rerun on the previously identified sequencing libraries. The GISTIC2.0 module, an evolution of the GISTIC (Genomic Identification of Significant Targets in Cancer) algorithm, identifies probable CNAs by evaluating the frequency and amplitude of observed events [27]. GISTIC was applied to several cancer types [28,29] and aided identifications of several new targets of amplifications and deletions [30,31], and thus was an ideal tool to provide quality metrics for confidence.

### 2.4. Statistical Analysis

Patient baseline clinical characteristics are presented as count and percentages and 95% confidence interval for proportion was calculated using exact method. The odds of docetaxel or cabazitaxel resistance (yes vs. no) for patients with *ABCB1* amplification or without were compared by odds ratio (OR). An OR greater than 1 indicates a higher likelihood of taxane resistance for patients with *ABCB1* amplification. OR and corresponding p-value were calculated using Firth’s bias-reduced logistic regression with profile penalized log-likelihood [32,33]. 

For the analysis of exploratory biomarkers, only amplifications or deletions with prevalence >10% (>4 and >2 patients in cohort A and B, respectively) were evaluated. ORs of taxane resistance for patients with an amplification (or deletion) vs. no amplification (or no deletion) were calculated and raw *p*-values for all observed gene aberrations were generated using Firth’s bias-reduced logistic regression with profile penalized log-likelihood and presented using volcano graphs (*x*-axis represents log(OR) and *y*-axis represents −log10(raw *p*-value)). To control for false discovery rate, *p*-values were adjusted by Benjamini–Yekutieli procedure. The biomarker was considered promising if the adjusted *p*-value was <0.05. 

## 3. Results

### 3.1. Cohorts

**Figure 1:**
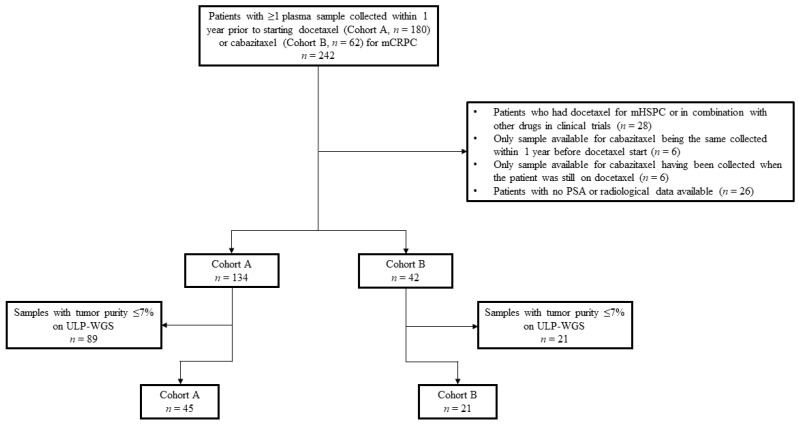
Schema of the clinical cohorts. Diagram describing reasons for excluding patients/samples from the initially 242 patients identified in the CRIS database. Abbreviations: mHSPC, metastatic hormone-sensitive prostate cancer; mCRPC, metastatic castration-resistant prostate cancer; PSA, prostate-specific antigen; ULP-WGS, ultra-low pass whole genome sequencing.

Of the 242 patients initially selected from the CRIS registry, 180 in cohort A and 62 in cohort B, 64 men were excluded from the study (Figure 1). Reasons for exclusion were the following: previous use of docetaxel for hormone sensitive disease or in combination with other drugs within clinical protocols, only plasma sample collected within 1 year prior to cabazitaxel start being the same available for docetaxel or drawn when the patient was still on docetaxel, and no PSA or radiological data available. Of the 176 remaining patients, 134 had at least one plasma sample collected and banked within 1 year prior to starting docetaxel and 42 prior to commencing cabazitaxel. Of these samples, ULP-WGS identified a total of 68 with sufficient tumor purity (>7%) to confidently detect *ABCB1* amplification and other CNAs. Because one patient in cohort A had three available samples with sufficient tumor content, the sample drawn closer to docetaxel start was prioritized and the other two were excluded. Thus, overall, 66 patients were eligible for this analysis: 45 patients in cohort A and 21 in cohort B. Four patients had one plasma sample available prior to docetaxel start and one prior to cabazitaxel initiation, and thus were counted in both cohorts.

### 3.2. Patient Characteristics

Patient clinical and radiological characteristics are described in Table 1. At the time of data annotation (May 2017), 98% of patients (44 of 45) in cohort A and 71% (15 of 21) in cohort B had died. More than half of men (51%; 23 of 45) in cohort A received 1–3 lines of therapy for mCRPC prior to starting docetaxel and 22% (10 of 45) had four or more previous treatments. Abiraterone acetate, enzalutamide, or radium-223 were not commonly administered before docetaxel. In cohort B, more than 2/3 of men (71%; 15 of 21) had progressed on at least four lines of therapy before receiving cabazitaxel; in particular, all patients had received docetaxel, 12 (57%) abiraterone acetate, and 7 (33%) enzalutamide. Most patients (53%; 24 of 45) in cohort A received at least six cycles of docetaxel. In cohort B, most patients (57%; 12 of 21) received less than four cycles of cabazitaxel. A PSA decline ≥ 50% was achieved in 42% (19 of 45) and 14% (3 of 21) of men in cohort A and B, respectively. Two of 45 men (4%) in the docetaxel cohort had a radiologic response within 4 months of the start of therapy, while none was observed in the cabazitaxel cohort. Primary resistance was observed in 26 of 45 patients (58%) in cohort A and in 18 of 21 (86%) in cohort B.

### 3.3. Laboratory and Clinical Outcomes

No statistically significant association was found between *ABCB1* amplification and primary resistance in cohort A (unadjusted *p* = 0.58; OR = 1.49, 95% CI: 0.36–7.11; Table 2) or B (unadjusted *p* = 0.97; OR = 1.06, 95% CI: 0.06–158.91; Table 3). The putative biomarker was observed in 9 of 45 patients (20.0%; 95% CI, 9.6–34.6) in cohort A and 6 of those (66.7%; 95% CI, 29.9–92.5) showed innate resistance to docetaxel. The rate of *ABCB1* amplification among patients with docetaxel innate resistance was 23.0% (95% CI, 9.0–43.7).

In cohort B, 2 of 21 patients (9.5%; 95% CI, 1.2–30.4) had *ABCB1* amplification prior to starting cabazitaxel and both of them showed primary resistance to cabazitaxel (Table 3). The rate of *ABCB1* amplification among the patients with cabazitaxel innate resistance was 11.1% (95% CI, 1.4–34.7).

**Figure 2:**
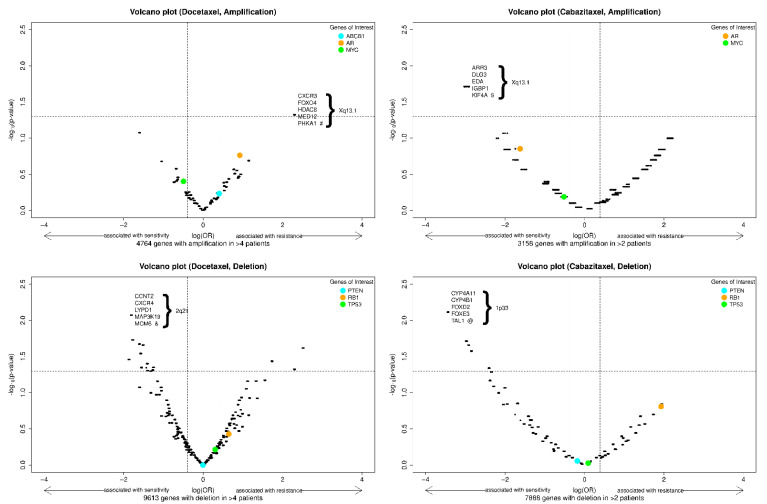
Volcano plots of exploratory biomarkers (amplifications, above; deletions, below) of resistance to docetaxel (**left**) or cabazitaxel (**right**). Note: *x*-axis = log(OR), *y*-axis = −log_10_(raw *p*-value). The vertical dash lines indicate an effect size (OR) of 2 and 0.5; the horizontal dash line indicates a (unadjusted) *p*-value of 0.05. The *p*-values are the raw *p*-value prior to false discovery rate adjustment. Specific genes of interest were indicated with colored dots. Among the genes with the most significant and identical *p*-values, a selected 5 are shown in each panel. #: 24 genes have most significant and identical *p*-values; $: 14 genes have most significant and identical *p*-values; &: 17 genes have most significant and identical *p*-values; @: 26 genes have most significant and identical *p*-values (Supplementary Table S1).

We also explored the association of genome-wide CNAs with sensitivity and resistance to docetaxel and cabazitaxel, as depicted in the volcano plots in Figure 2. This analysis did not identify any CNAs (amplification or deletion) predictive of primary resistance to docetaxel or cabazitaxel after adjusting for the false discovery rate. Interestingly, in this cohort, the amplification of the *AR* gene locus trended towards association with resistance to docetaxel and sensitivity to cabazitaxel, while *RB1* deletion trended towards association with resistance to both agents. The amplified gene segment most highly associated with docetaxel resistance was in chromosome *Xp13.1*, and the deleted segment most highly associated with docetaxel sensitivity was in chromosome *2q21*. The amplified gene segment most highly associated with cabazitaxel sensitivity was in chromosome *Xp13.1*, and the deleted gene segment most highly associated with cabazitaxel sensitivity was in chromosome *1p33*. Genes of interest within these segments are depicted in the figure, and a list of genes with the smallest and identical p-values classified by docetaxel or cabazitaxel cohort and type of CNA is reported in Table S1 of the Supplementary Information.

## 4. Discussion

Although several new treatment options have recently been approved for mCRPC, docetaxel and cabazitaxel remain the standard chemotherapeutics for this state of disease. Despite their demonstrated efficacy for most patients with mCRPC, there is a portion of men who have primary resistance to these agents [3,4,5]. In the past, several studies aimed to correlate biological or genetic features in prostate cancer with resistance to taxane-based therapy [7,8,9,10]. However, no biomarker of resistance to taxane-based therapy has been validated. Based on existing evidence for the role of ATP-binding cassette (ABC) transporters in taxane resistance, we sought to assess the correlation between the putative biomarker *ABCB1* gene amplification or other CNAs detected through ULP-WGS used on plasma-extracted cfDNA and innate resistance to docetaxel or cabazitaxel for mCRPC. 

No statistically significant association was observed between the putative biomarker or any other exploratory CNA and primary resistance to docetaxel or cabazitaxel in this study. This analysis was underpowered due to the relatively small sample size (45 patients for A and 21 for B) resulting from the requirement for selecting specimens from the original population (*n* = 176) with sufficient tumor purity (>7%) for reliable output quality. The rates of samples with tumor-derived cfDNA >7% detected in cohort A (33%) and in cohort B (50%) are consistent with our previous report, where tumor fraction in cfDNA >10% was found in ~29% of mCRPC patients [23]. This requirement does limit the applicability of this method in patients with lower tumor fraction. 

For the docetaxel cohort, given the sample size in this analysis (*n* = 45), prevalence of ABCB1 amplification (20%), and resistance observed in these patients (*n* = 26, 58%), the maximum power attainable for putative effect is about 60% with a detectable odds ratio of more than eight (assuming no multiple comparison adjustment). For the cabazitaxel cohort, given the sample size in this analysis (*n* = 21), prevalence of ABCB1 amplification (10%), and resistance observed in these patients (*n* = 18, 86%), the maximum power attainable is about 30% with a protective OR of 0.1. Given the modest prevalence of ACBC1 amplification (20%) and high proportion of resistance (58%), a sample size around 100 will provide an 80% power to detect an OR of 9.5, and a sample size around 200 will provide an 80% power to detect an OR of 3.71. As such, the data we present here are intended to provide a proof of principle for the techniques described that copy number profiling from cfDNA is feasible and potentially clinically relevant in the subset of patients with high tumor fraction. Future studies with a larger sample size are warranted to achieve proper statistical power to test whether ABCB1 amplification or the copy number alterations depicted in Figure 2 are predictive for taxane resistance or response. 

Another limitation of this study is that, due to sparse sequencing and the requirement for binning over 1 Mb segments, ichorCNA can identify CNAs over large genomic regions but would not detect more focal amplification events. Additionally, this method is intended to detect the amplification of the *ABCB1* gene locus and would not be able to identify overexpression of the ABCB1 protein through non-genetic mechanisms. Of note, *ABCB1* amplification was observed more frequently in cohort A (20.0%) and B (9.5%) compared to what has been previously reported in the Stand Up 2 Cancer database (~2%). This could be due to different methodologies or thresholds for calling amplifications, the tumor fraction >7% cut-off selecting for patients more likely to have *ABCB1* amplification, or our study population reflecting a more heavily pre-treated cohort [34,35,36]. We are currently in the process of performing deep targeted-sequencing of the same cfDNA libraries achieved in the present analysis, which would permit the detection of a variety of genetic alterations, including more focal *ABCB1* amplification as well as the other putative biomarkers *KDM5D* deletion and *ERG-TMPRSS2* translocation, even in cfDNA samples with tumor purity <7%. 

Despite the limitations of our analysis, our observations that patients with *ABCB1* amplification have a higher frequency of innate resistance to docetaxel (66.7% vs. 57.8% in the overall population) and cabazitaxel (100% vs. 85.7%) are promising data, which warrant further investigation in a larger dataset. Future analyses demonstrating *ABCB1* amplification as a biomarker of primary resistance to either taxane for mCRPC would allow for the consideration of alternative treatment strategies, including the selection of patients for co-administration of an inhibitor of ABCB1/P-glycoprotein. These inhibitors have shown promising activity in reversing taxane resistance in vitro [37,38,39], and ritonavir is being used as a P-glycoprotein inhibitor in combination with oral docetaxel to increase the bioavailability of the taxane [40]. Because prior clinical trials of ABCB1/P-glycoprotein inhibitors have demonstrated notable toxicities, while novel inhibitors are in development [41], the ability to select for patients most likely to benefit from these agents is potentially clinically relevant. 

Our analysis also nominates novel amplification and deletion events for validation of association with docetaxel and cabazitaxel sensitivity and resistance in larger datasets. Banked plasma is generally more readily available than metastatic tissue specimens for retrospective analyses, so the expansion of these studies using frozen plasma from existing biospecimen banks is feasible. Further, because obtaining cfDNA is certainly less invasive than achieving tumor biopsy specimens, and ULP-WGS is a much less expensive technique than deep sequencing from cfDNA or the analysis of circulating tumor cells, the present analysis could pave the way to larger studies using this method to investigate biomarkers predictive of taxane therapy, which, if validated, could have the potential to be more easily accessible and broadly used in clinical practice. 

## 5. Conclusions

In a small sample of patients treated with docetaxel or cabazitaxel for mCRPC, no significant association was observed between the putative biomarker *ABCB1* amplification, identified using ULP-WGS on plasma-derived cfDNA, and primary resistance to docetaxel or cabazitaxel. However, data were promising and future analyses using deeper genomic profiling or with larger datasets may aid in drawing definitive conclusions. 

## Figures and Tables

**Table 1:** Patient characteristics.

Variables	Cohorts	
	Docetaxel (A) *n* = 45	Cabazitaxel (B) *n* = 21
Still alive, *n* (%)		
No	44 (98)	15 (71)
Yes	1 (2)	6 (29)
Prior treatments for mCRPC, *n* (%)		
0	12 (27)	0 (0)
1–3	23 (51)	6 (29)
≥4	10 (22)	15 (71)
Prior abiraterone acetate, *n* (%)		
No	39 (87)	9 (43)
Yes	6 (13)	12 (57)
Prior enzalutamide, *n* (%)		
No	43 (96)	14 (67)
Yes	2 (4)	7 (33)
Prior radium-223, *n* (%)		
No	44 (98)	19 (90)
Yes	1 (2)	2 (10)
Prior docetaxel, *n* (%)		
No	45 (100)	0 (0)
Yes	0 (0)	21 (100)
Resistance, *n* (%)		
No	19 (42)	3 (14)
Yes	26 (58)	18 (86)
PSA decline ≥ 80% within 4 months from taxane start, *n* (%)		
No	36 (80)	20 (95)
Yes	9 (20)	1 (5)
PSA decline ≥ 50% within 4 months from taxane start, *n* (%)		
No	26 (58)	18 (86)
Yes	19 (42)	3 (14)
Radiological response within 4 months from taxane start, *n* (%)		
No	42 (93)	21 (100)
Yes	2 (4)	0 (0)
N/A	1 (2)	0 (0)
Cycles of taxane, *n* (%)		
1–3	12 (27)	12 (57)
4–6	4 (9)	2 (10)
>6	24 (53)	7 (33)
N/A	5 (11)	0 (0)

Abbreviations: mCRPC, metastatic castration-resistant prostate cancer; N/A, not available; PSA, prostate-specific antigen.

**Table 2:** Association between ABCB1 amplification and primary resistance to docetaxel in cohort A (*n* = 45).

	ABCB1 Amplification, *n* (%)	No ABCB1 Amplification, *n* (%)	Total, *n* (%)
Resistance, *n* (%)	6 (13.3)	20 (44.5)	26 (57.8)
No resistance, *n* (%)	3 (6.7)	16 (35.5)	19 (42.2)
Total, *n* (%)	9 (20.0)	36 (80.0)	45 (100.0)

*p*-value = 0.58; Odds Ratio = 1.49 (95% CI: 0.36–7.11); *p*-value and odds ratio were calculated using Firth’s bias-reduced logistic regression.

**Table 3:** Association between ABCB1 amplification and primary resistance to cabazitaxel in cohort B (*n* = 21).

	ABCB1 Amplification, *n* (%)	No ABCB1 Amplification, *n* (%)	Total, *n* (%)
Resistance, *n* (%)	2 (9.5)	16 (76.2)	18 (85.7)
No resistance, *n* (%)	0 (0.00)	3 (14.3)	3 (14.3)
Total, *n* (%)	2 (9.5)	19 (90.5)	21 (100.0)

*p*-value = 0.97; Odds Ratio = 1.06 (95% CI: 0.06–158.91); *p*-value and odds ratio were calculated using Firth’s bias-reduced logistic regression.

## Data Availability

Almost all data are included in the manuscript and in the Supplementary Information. However, additional data are available from the corresponding author upon reasonable request.

## Supplementary Materials

The following are available online at https://www.mdpi.com/article/10.3390/cancers13164055/s1, Table S1: Genes with the most significant and identical *p*-values.
