# Supplementary material for: Circulating Cell-Free DNA as Biomarker of Taxane Resistance in Metastatic Castration-Resistant Prostate Cancer
Source: Cancers (Basel). 2021 Aug 12;13(16):4055. doi: 10.3390/cancers13164055 (PMC8391478; doi:10.3390/cancers13164055)
Supplement: Supplementary file 1 [file cancers-13-04055-s001.zip › cancers-1328462-supplementary.pdf]

cancers

MDPI

Supplementary Materials

Circulating Cell-Free DNA as Biomarker of Taxane Resistance in Metastatic Castration-Resistant Prostate Cancer

Edoardo Francini, Fang-Shu Ou, Justin Rhoades, Eric G. Wolfe, Edward P. O'Connor, Gavin Ha, Gregory Gydush, Kaitlin M. Kelleher, Rupal S. Bhatt, Steven P. Balk, Christopher J. Sweeney, Viktor A. Adalsteinsson, Mary-Ellen Taplin and Atish D. Choudhury

Table S1. Genes with the most significant and identical p-values.

Docetaxel, Amplification (raw p-value*=0.048)			Cabazitaxel, Amplification (raw p-value*=0.019)	
ACRC	ITGB1BP2	SLC7A3	ARR3	P2RY4
CITED1	MED12	SNX12	AWAT1	PDZD11
CXCR3	NHSL2	TAF1	AWAT2	RAB41
CXorf65	NLGN3	ZMYM3	DGAT2L6	TEX11
DMRTC1B	NONO		DLG3	
ERCC6L	OGT		EDA	
FOXO4	PHKA1		GDPD2	
GJB1	PIN4		IGBP1	
HDAC8	RGAG4		KIF4A	
IL2RG	RPS4X		OTUD6A	

Docetaxel, Deletion (raw p-value*=0.0085)		Cabazitaxel, Deletion (raw p-value*=0.0077)		
ACMSD	MGAT5	AGBL4	DMRTA2	SLC5A9
ANKRD30BL	NCKAP5	AL109659.1	EFCAB14	SPATA6
CCNT2	R3HDM1	ATPAF1	ELAVL4	STIL
CXCR4	RAB3GAP1	BEND5	FLJ00388	TAL1
DARS	TMEM163	CMPK1	FOXD2	TEX38
GPR39	UBXN4	CYP4A11	FOXE3	TRABD2B
LCT	ZRANB3	CYP4A22	KNCN	
LYPD1		CYP4B1	MKNK1	
MAP3K19		CYP4X1	MOB3C	
MCM6		CYP4Z1	PDZK1IP1	

*: p-value was calculated using Firth's biased-reduced logistic regression prior to false discovery rate adjustment.
